# Insular and Hippocampal Gray Matter Volume Reductions in Patients with Major Depressive Disorder

**DOI:** 10.1371/journal.pone.0102692

**Published:** 2014-07-22

**Authors:** Mirjam Stratmann, Carsten Konrad, Harald Kugel, Axel Krug, Sonja Schöning, Patricia Ohrmann, Christina Uhlmann, Christian Postert, Thomas Suslow, Walter Heindel, Volker Arolt, Tilo Kircher, Udo Dannlowski

**Affiliations:** 1 Department of Psychiatry, University of Marburg, Marburg, Germany; 2 Department of Psychiatry, University of Münster, Münster, Germany; 3 Department of Clinical Radiology, University of Münster, Münster, Germany; 4 Department of Child and Adolescent Psychiatry, Psychosomatics and Psychotherapy, University of Münster, Münster, Germany; 5 Department of Psychosomatic Medicine and Psychotherapy, University of Leipzig, Leipzig, Germany; Centre Hospitalier Universitaire Vaudois Lausanne - CHUV, UNIL, Switzerland

## Abstract

**Background:**

Major depressive disorder is a serious psychiatric illness with a highly variable and heterogeneous clinical course. Due to the lack of consistent data from previous studies, the study of morphometric changes in major depressive disorder is still a major point of research requiring additional studies. The aim of the study presented here was to characterize and quantify regional gray matter abnormalities in a large sample of clinically well-characterized patients with major depressive disorder.

**Methods:**

For this study one-hundred thirty two patients with major depressive disorder and 132 age- and gender-matched healthy control participants were included, 35 with their first episode and 97 with recurrent depression. To analyse gray matter abnormalities, voxel-based morphometry (VBM8) was employed on T1 weighted MRI data. We performed whole-brain analyses as well as a region-of-interest approach on the hippocampal formation, anterior cingulate cortex and amygdala, correlating the number of depressive episodes.

**Results:**

Compared to healthy control persons, patients showed a strong gray-matter reduction in the right anterior insula. In addition, region-of-interest analyses revealed significant gray-matter reductions in the hippocampal formation. The observed alterations were more severe in patients with recurrent depressive episodes than in patients with a first episode. The number of depressive episodes was negatively correlated with gray-matter volume in the right hippocampus and right amygdala.

**Conclusions:**

The anterior insula gray matter structure appears to be strongly affected in major depressive disorder and might play an important role in the neurobiology of depression. The hippocampal and amygdala volume loss cumulating with the number of episodes might be explained either by repeated neurotoxic stress or alternatively by higher relapse rates in patients showing hippocampal atrophy.

## Introduction

Major depressive disorder (MDD) is an affective disorder causing significant disability and suffering. With a lifetime prevalence of about 16% [1] it is the most prevalent mood disorder and one of the leading causes of burden of disease [2].

The neuroanatomical substrates of major depression are so far inconsistent and still under debate. To understand the neurobiological underpinnings of major depression and recurrent MDD in particular, brain morphological alterations have been repeatedly studied, using MRI and voxel-based morphometry (VBM) techniques. However, results vary considerably across studies [3]–[5]. According to recent meta-analysis from Lai et al. [6] and Bora et al. [3] gray matter deficits of the anterior cingulate cortex rank among the most consistent findings in VBM studies of MDD. Other frequently reported abnormalities include volumetric reductions in the frontal cortex, orbitofrontal cortex, ventral striatum, thalamus and hippocampus [3]–[5], [7], [8]. Of these structures, morphometric reduction in the hippocampus is one of the most replicated finding described in the literature in MDD, with both a ROI approach [4] and VBM [9]–[11]. The reported regions are part of an extended network including the medial prefrontal cortex and anatomically related limbic, striatal, thalamic and basal forebrain structures [12]. The structural imaging abnormalities found in MDD have been associated with histopathological abnormalities in post mortem studies [12]. Bielau et al. [13] reported gray matter volume reduction of the external pallidum, hypothalamus, amygdala, basal limbic forebrain and nucleus accumbens bilaterally. Stockmeier et al. [14] reported significant reduction in neuropil in the hippocampus in MDD, which may account for decreased hippocampal volume detected by neuroimaging.

However, despite the existing wealth of structural neuroimaging studies on depression, discrepant results were described across studies concerning the limbic system, conceivably reflecting clinical and etiological heterogeneity extant within the MDD syndromes [12]. According to the meta-analysis of Bora et al. [3] 25–50% of the studies reported gray matter abnormalities of right fronto-insula cortex. The insula cortex, with its extensive connections to fronto-limbic regions, seems to be an important part of this network [15]. Insular gray-matter reductions are frequently reported in studies of MDD [3], [16]–[19], but results differ, for example in laterality and concerning the interpretation of the influence of the course of disease.

Reasons for inconsistent or even divergent results are complex. One major problem might be the heterogeneity of studies regarding important clinical variables, of which the neurostructural correlates are barely understood hitherto, and which have rarely been investigated in subgroups within the same study due to the small sample sizes in most of the studies. E.g., some studies investigated only MDD patients with first depressive episode [11], [19]–[23], while others included patients with recurrent episodes [17], [24]–[29]. MDD samples also varied largely regarding illness-duration, number of depressive episodes or illness-onset. Depression severity of MDD adds further variance to study results, with some studies investigating only acute patients while others also included remitted patients [29]–[32]. The morphological correlates of sub-groups of MDD also attracted scientific interest, for example MDD patients with comorbid panic or anxiety disorder [21], [33] or MDD patients with melancholic depression [17]. Studies also differed notably in age-composition with mean-ages varying from ≤30 years [20], [22], [23], [34] to >60 years [17], [27]. The relevance of these clinical variables was demonstrated by a review of Schindler et al. [35] and meta-analyses of Lai et al. [6] and Bora et al. [3]. E.g., older age, male sex as well as the lack of medication was correlated with ACC gray matter deficits. Other structures were related to current depression severity and duration of illness [6]. While patients in multi-episode samples had decreased gray matter in ACC and dorsomedial frontal cortex compared to controls, first-episode patients had a significant gray matter reduction in right superior temporal gyrus, parahippocampal gyrus and amygdala [3]. Patients with co-morbid anxiety disorder had significantly less gray matter in the right amygdala/parahippocampal gyrus extending to the putamen [3]. In addition to these obviously important clinical factors, there were considerable methodological differences between the studies concerning MRI data acquisition, most obviously between region-of-interest (ROI) and whole brain analysis, but also concerning processing steps and statistical analysis, e.g. different types of correction for multiple comparisons [36]. Furthermore, many studies were limited by small sample sizes, including less than twenty MDD patients [21], [22], [24], [25].

The aim of the study presented here was to characterize and quantify regional gray matter abnormalities in a large sample of clinically well-characterized patients with major depressive disorder by using voxel-based morphometry (VBM).

As one particularity of this study, analysis of patient sub-groups, e.g. patients with first depressive episodes and patients with recurrent episodes, was performed.

Using state-of-the-art 3T-MR imaging, in the first step, gray-matter reductions in patients with MDD in comparison to healthy control subjects were investigated in an exploratory whole-brain analysis. According to the literature, we hypothesize, that there are widespread gray matter volume reductions in patients with MDD, involving structures of the mentioned networks. We hypothesized, that gray matter volume reductions were stronger in patients with a long duration of illness.

Because of inconsistent results of previous studies, we were especially interested in gray matter alterations of the limbic system. Therefore, in the next step, the role of the limbic system was prompted using ROI-analyses on pre-defined limbic regions (hippocampus, amygdala). According to the literature we hypothesize, that significant structural changes will occur in the hippocampus in patients with first depressive episode and in patients with recurrent depressive episodes. We further hypothesize, that structural changes will occur in the amygdala in patients with first and recurrent depressive episodes. We hypothesize, that gray matter volume reductions in hippocampus and amygdala were stronger in patients with a long duration of illness.

In further post-hoc analyses, we explored the influence of severity of depressive symptoms (BDI, HDRS), comorbid anxiety disorder, and possible influences of medication on regional gray matter volume in exploratory whole-brain analyses.

## Methods and Materials

### Subjects

One-hundred thirty two patients with MDD and 132 healthy control participants were recruited at the University Hospital of Münster from 2005 to June 2011. MDD and control groups were matched concerning age and gender (Table 1). The patients' ages ranged from 18 to 60 years. All participants were right-handed according to the Edinburgh Handedness Inventory [37].

**Table 1:** Sociodemographic characteristics of the whole sample.

Comparison: Controls with	Category	Patients	Controls	Statistics *	
**All patients**	n	132	132		
	Age in years (SD)	37.86 (11.87)	37.82 (11.42)	t = −0.026	p = 0.979
	Female/male	76/56	74/58	x^2^ = 0.062	p = 0.804
	MWT-B (SD)	112.02 (13.42)	118.43 (11.52)	t = 4.163	p = 0.001
**Patients with first depressive episode**	n	35	35		
	Age in years (SD)	34.86 (11.69)	35.14 (11.14)	t = 0.105	p = 0.917
	Female/male	21/14	19/16	x^2^ = 0.233	p = 0.629
	MWT-B (SD)	110.06 (11.96)	117.69 (11.27)	t = 2.801	p = 0.007
**Patients with recurrent depressive episodes**	n	97	97		
	Age in years (SD)	38.94 (11.81)	38.78 (11.41)	t = −0.093	p = 0.926
	Female/male	55/42	55/42	x^2^ = 0.000	p = 1.000
	MWT-B (SD)	112.73 (13.89)	118.70 (11.81)	t = 3.223	p = 0.001

SD  =  standard deviation.

MWT-B: Multiple-choice vocabulary test.

* Group differences were computed using independent sample t-test for continuous and Chi-square-test for categorial variables. The level of statistical significance was set at p<0.05.

MDD participants were assessed by an experienced psychiatrist and met criteria for diagnosis of DSM-IV MDD. Inclusion criteria were diagnosis of either first (n = 35) or recurrent episode (n = 97) of unipolar depression, verified by the standardized SCID-I Interview (Structured Clinical Interview for DSM-IV; German version, [38]). Patients with a history of hypomanic and manic episodes as well as patients with comorbid alcohol or substance abuse (life time diagnosis) were excluded from this study. Anxiety disorder as comorbidity was not an exclusion criteria provided that it was not the primary reason for current hospitalization. Forty-one patients with a comorbid anxiety disorder were included (Table 2). Patients with further comorbid axis-I and axis-II disorders were excluded from the study. Serious head injury in the past, past or current serious medical or neurological disease, neurodegenerative diseases as well as MRI contraindications were further exclusion criteria. None of the patients had a history of electroconvulsive therapy in the past. One-hundred twenty-six patients were treated according to current treatment guidelines [39] and six patients did not receive any medication. The following antidepressants were prescribed as antidepressive monotherapy (n = 76), antipsychotic monotherapy (n = 2), combined antidepressive therapy (n = 22), or combined antidepressive/antipsychotic therapy (n = 26): selective serotonin-noradrenaline-reuptake-inhibitors (SSNRI) (n = 62), Mirtazapine (n = 44), selective serotonin-reuptake-inhibitors (SSRI) (n = 37), selective noradrenaline-reuptake-inhibitors (SNRI) (n = 6), tricyclic antidepressants (n = 4), Agomelatine (n = 3), Bupropion (NDRI) (n = 2), atypical antipsychotics (n = 27). Eight patients were taking lithium alone or in combination with other antidepressive or antipsychotic medication; five patients were taken mood stabilizers (Lamotrigine (n = 4), Topiramate (n = 1)). None of the patients was taking benzodiazepines at the time of testing. (Details concerning medication: Table 2 and Table S1).

**Table 2:** Clinical characteristics of the patients' sample (n = 132).

	MDD first depressive episode n = 35	MDD recurrent depressive episodes n = 97	Statistics*	
**Age in years (SD)**	34.86 (11.69)	38.94 (11.81)	t = −1.758	p = 0.081
**Female/male**	21/14	55/42	x^2^ = 0.115	p = 0.735
**Verbal IQ (MWT-B)**	110.06 (11.96)	112.73 (13.89)	t = −1.011	p = 0.314
**Number of depressive episodes**	1	4,9 (4,5); 2–20		
**Time since diagnosis (months)**	14.66 (15.73)	121.75 (109.64)		
**HDRS mean (standard deviation)**	19,46 (9,94)	20,85 (8,10)	t = −0.821	p = 0.413
**BDI mean (standard deviation)**	21,06 (10,71)	22,83 (11,13)	t = −0.799	p = 0.426
**Comorbidities**			x^2^ = 0.003	p = 0.956
No comorbidities	24	67		
Comorbidities: anxiety disorders	11	30		
**Medication**			x^2^ = 1.415	p = 0.842
No medication	2	4		
Antidepressive monotherapy	22	54		
Antipsychotic monotherapy	0	2		
Combined antidepressive therapy	5	17		
Combined antidepressive/antipsychotic therapy	6	20		

MDD: major depressive disorder.

SD  =  standard deviation.

MWT-B: Multiple-choice vocabulary test.

HDRS: Hamilton Depression Rating Scale.

BDI: Beck's Depression Inventory.

* Group differences were computed using independent sample t-test for continuous and Chi-square-test for categorial variables. The level of statistical significance was set at p<0.05.

One-hundred thirty-two control persons, belonging to the same sociodemographic environment as the patients were recruited by advertisement in the local newspaper. All control subjects underwent an initial telephone screening to ensure matching criteria, and received a full SCID-interview to exclude psychiatric diagnoses. Further exclusion criteria were neurological and neurodegenerative diseases, serious medical diseases, any psychotropic medication as well as MRI contraindications.

Verbal intelligence was estimated using the multiple-choice vocabulary test (MWT-B) [40]. Current depression severity was assessed by means of the Hamilton Depression Rating Scale (HDRS) [41] and Beck Depression Inventory (BDI) [42].

### Ethics Statement

The study was conducted in accordance with the Declaration of Helsinki. Approval was obtained from the ethics committee at the University of Münster. After a comprehensive description of the study to the participants, written informed consent was obtained.

### MRI data acquisition

MRI data acquisition was performed in a 3 Tesla whole-body scanner (Intera T 3.0, Philips, Best, NL). A circularly polarized transmit/receive birdcage head coil with an HF reflecting screen at the cranial end was used for spin excitation and resonance signal acquisition. For each participant, a T1 structural MRI image was acquired using a 3D fast gradient echo sequence (‘Turbo Field Echo', TR = 7.4 ms, TE 3.4 ms, FA = 9°, 2 signal averages, inversion prepulse every 814.5 ms, acquired over a field of view of 256 (FH)×204 (AP)×160 (RL) mm, phase encoding in AP and RL direction, reconstructed to cubic voxels of 0.5 mm edge length). All MRI images were visually inspected for artifacts and anatomical abnormalities as well as neurodegenerative changes.

There was an upgrade of the scanner gradient system in 2008 (“Master” Gradient System to “Quasar Dual” Gradient System). 69 patients and 50 controls were included before and 63 patients and 82 controls were included after the gradient system update. Albeit the MRI sequence remained identical before and after the gradient system upgrade, we additionally modeled the scanner update as covariate of no interest in the second level SPM-analysis.

Concerning this scanner gradient update, reliability tests demonstrated high test-retest correlations: We analyzed scans of healthy subjects (not included in the present study) who have been scanned twice, either with both scans conducted after the upgrade (N = 5) or one scan conducted before and the other conducted after the upgrade (N = 3), with several months between the two scans in both groups. Furthermore, a subsample of depressed patients included in the present study was scanned twice about seven weeks apart (N = 9). All MRI scans were conducted with the sequence used in our manuscript. We employed the “check homogeneity using covariance” function within the VBM8-toolbox for correlating gray matter segments from the T1-images with those from the same subjects at the second time point as a measure for test-retest reliability (rtt). The test-retest correlations were very high for all these participants (all rtt>.93). The mean correlation in the group of healthy subjects with two scans after the gradient upgrade was 0.953 and for the group of subjects with one scan before and the other scan after the upgrade, it was 0.958, which is practically identical. The figure was 0.960 for the patients scanned seven weeks apart, probably reflecting the much shorter period between the two time points.

### Voxel-based morphometry

Data were preprocessed using the VBM8-toolbox (http://dbm.neuro.uni-jena.de/vbm.html). Default settings were used. Structural images were bias-corrected, tissue-classified and normalized to Montreal Neurological Institute space using linear (12-parameter affine) and nonlinear transformations, within a unified model [43] including high-dimensional DARTEL normalization. Gray-matter volume per voxel was calculated by applying an absolute threshold masking of 0.2 and modulating the normalized segmented images with a non-linear only warping. This results in an analysis of relative differences in regional gray-matter volumes, which are corrected for different brain sizes. To check the quality of the segmentation and normalization procedures, the normalized, bias-corrected images were visually inspected. In addition, covariance between normalized segmented images was calculated to check homogeneity of variance and to identify potential outliers. MRI images with artifacts, anatomical abnormalities as well as neurodegenerative changes were excluded from this study. Finally the normalized, segmented and modulated volumes were smoothed with an 8 mm full width at half maximum (FWHM) Gaussian kernel.

### Statistical analyses

Statistical analyses of the structural MRI images were conducted using 2^nd^ level models implemented in SPM8 software (Wellcome Department of Imaging Neuroscience Group, London, UK; http://www.fil.ion.ucl.ac.uk/spm). Group statistics were calculated using independent sample t-tests, correlation analyses were conducted using multiple regression analyses. Whole brain analyses as well as region-of interest (ROI) approaches were conducted. Time of gradient upgrade, age, sex and MWT-B [40] were entered as covariates of no interest in all analyses.

To control for multiple statistical testing within the entire brain, we maintained a cluster-level false-positive detection rate at p<0.05 using a voxel-level threshold of p<0.001 with a cluster extend (k) empirically determined by Monte Carlo simulations (n = 1000 iterations), by means of AlphaSim procedure [44], implemented in the REST toolbox (http://www.restfmri.net/forum/REST_V1.7) [45]. AlphaSim procedure is a well-accepted and established approach to control for multiple statistical testing determined by Monte Carlo simulations within the entire brain as well as in ROI-analyses [44], [46]. The empirically determined cluster threshold for whole brain analyses was k = 139 voxels. The anatomical labelling of the identified cluster regions were done by reference to the AAL-definitions [47].

According to previously described affected brain regions in patients with MDD we defined the following ROIs using the AAL-definitions [47]: hippocampus + parahippocampal gyrus, amygdala and ACC, each bilaterally. ROI-Masks were created by means of the Wake Forest University (WFU) PickAtlas toolbox, Version 2.5.2. (http://fmri.wfubmc.edu/cms/software). To control for multiple statistical testing in ROI-analyses, we performed the AlphaSim procedure as described above. In comparison to whole brain analyses, we used a voxel-level threshold of p<0.01. The empirically determined clusters thresholds were k = 109 for the bilateral hippocampus+parahippopcampal gyrus mask, k = 10 for the bilateral amygdala mask and k = 120 for the bilateral ACC mask.

In a first step, we compared structural gray-matter volume differences between all patients (n = 132) and all healthy control subjects (n = 132) participating in the study. To evaluate possible differences between patients with first episode of MDD and patients with recurrent episodes, as compared to healthy control subjects, we further compared patients with first episodes (n = 35) versus controls (n = 35) and patients with recurrent episodes (n = 97) versus controls (n = 97). In all analyses, MDD and control samples were matched according to age and gender. Time of gradient upgrade, age, sex and MWT-B [40] were entered as covariates of no interest in all multiple regression analyses. AlphaSim procedure was performed to control for multiple statistical testing in all multiple regression analyses as described above.

In addition, we also compared patients with first depressive episode and patients with recurrent depressive episodes.

To assess the correlation between regional gray-matter volume and number of depressive episodes in patients with MDD (n = 132) we performed whole brain multiple regression analyses. To access specifically the influence of number of depressive episodes on hippocampus, amygdala and ACC, we performed multiple regression analyses in predefined regions of interest as described above. Time of gradient upgrade, age, sex and MWT-B [40] were entered as covariates of no interest in all multiple regression analyses. AlphaSim procedure was performed to control for multiple statistical testing in all multiple regression analyses as described above.

In further post-hoc analyses, we explored the influence of severity of depressive symptoms (BDI, HDRS), comorbid anxiety disorder, and possible influences of medication on regional gray matter volume. To prevent possible influences of lithium-medication, we repeated the described whole brain group analyses in the same way after excluding patients with a lithium-medication (n = 8). Furthermore, we performed a whole brain group analysis comparing patients with (n = 28) and without (n = 104) antipsychotic medication to evaluate possible influences of antipsychotics on gray matter volume. To evaluate possible differences between patients with co-morbid anxiety disorder (n = 41) and patients without co-morbidity we performed a whole brain group analyses. We explored the influence of severity of depressive symptoms by performing whole brain multiple regression analyses both with BDI and HAMD as additional covariates. Time of gradient upgrade, age, sex and MWT-B [40] were entered as covariates of no interest in all described analyses. AlphaSim procedure was performed to control for multiple statistical testing as described above.

## Results

### Demographic data and clinical characteristics of the subjects

In order to ensure comparability between patients and control persons, age ranges as well as gender ratios were balanced (Table 1). We found that in the overall sample patients with MDD had significant lower verbal intelligence (MWT-B) values than healthy controls (Table 1). Therefore, MWT-B was included as covariate.

Comparing patients with first depressive episode (n = 35) and patients with recurrent depressive episodes (n = 97), there were no significant differences according to age, gender and MWT-B. The average number of depressive episodes of all patients was 3.9 (4.2; range: 1–20). The average duration of the current depressive episode (n = 71) was 29.31 weeks (range: 1–144, SD: 33.12). Cumulative duration of depressed episodes (n = 75) was 28.09 months (range: 0–180, SD: 31.95). The average time since diagnosis (n = 132) was 93.36 months (range: 0–564, SD: 105.49). The average HDRS and BDI scores for depressed patients were 20.48 (8.61) and 22.35 (11.01) respectively. There were no significant differences according to acuity, comorbid anxiety disorder and medication between the patients groups (Table 2).

### VBM-analyses: regional gray matter alterations in MDD versus control participants

There were no significant differences in global gray-matter volume (MDD: 625.75 (Standard deviation (SD): 58.99), controls: 629.27 (SD: 54.37), t = .505, p = .614), global white matter volume (MDD: 518.00 (SD: 56.71), controls: 513.92 (SD: 61.19), t = −.561, p = .575) and total brain volume (MDD: 1143.75 (SD: 106.39), controls: 1143.19 (SD: 108.95), t = −.041, p = .697) between patients and controls.

#### Whole-brain analyses

**Figure 1:**
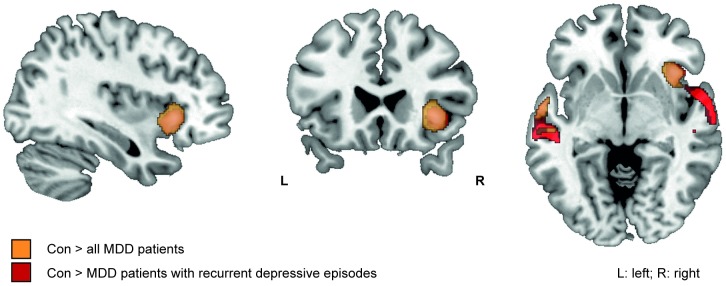
Gray matter volume reductions in whole brain analysis. Gray matter volume reductions in all MDD patients versus healthy controls (orange), and patients with recurrent depressive episodes versus healthy controls (red) (Table 3). (Whole brain analyses, p<0.001, k = 139; view: MNI: 36 23 -5).

According to our hypothesis, we found widespread gray matter volume reductions in patients with MDD. Compared to healthy control participants, patients with MDD showed significant gray-matter reductions in the right anterior insula, superior temporal gyrus bilaterally, left superior parietal gyrus and left parahippocampal gyrus. (Table 3, Figure 1). We could not find significant gray matter deficits in the anterior cingulate cortex as well as in the frontal cortex, orbitofrontal cortex, ventral striatum and thalamus.

**Table 3:** Results of whole brain voxel-based morphometry (VBM) analyses.

Contrast	Anatomical region	Side	Cluster size	MNI Coordinates (mm)			Z-Score	p-value (uncorr.)
				x	y	z		
**A) Con>Pat**	Insula	R	775	36	23	−5	4.47	<0.001
	SPL	L	393	−24	−72	45	4.36	<0.001
	STG	L	757	−60	−1	−3	3.98	<0.001
	STG	R	338	56	−7	−12	3.94	<0.001
	Parahippocampal Gyrus	L	446	−27	−31	−20	3.80	<0.001
**B) Con>Pat with first depressive episode**	No significant differences found in any region							
**C) Con>Pat with rec. depressive episodes**	SPL	L	434	−24	−72	46	4.47	<0.001
	MTG	L	1224	−62	−10	−9	4.40	<0.001
	STG	R	945	60	−7	−11	4.32	<0.001
	Insula	R	470	38	23	−5	4.29	<0.001
	MTG	R	174	52	−69	4	3.48	<0.001

Contrasts: A) all MDD patients (n = 132) versus healthy controls (n = 132), B) patients with first depressive episode (n = 35) versus healthy controls (n = 35), C) patients with recurrent depressive episodes (n = 97) versus healthy controls (n = 97). Analyses where conducted at p<0.001, uncorrected, k = 139 voxels.

MDD: major depressive disorder.

Con: controls; Pat: patients.

STG: superior temporal gyrus.

SPL: superior parietal lobule.

MTG: middle temporal gyrus.

L: left; R: right.

According to our hypothesis, gray matter volume reductions were stronger in patients with a long duration of illness. There were no differences in regional gray-matter volumes between patients with first depressive episodes and healthy control subjects (Table 3, Figure 1). Patients with recurrent depressive episodes showed significant gray-matter reductions in the right anterior insula, right superior temporal gyrus, bilateral middle temporal gyrus and left superior parietal gyrus compared to healthy control subjects (Table 3, Figure 1).

In contrast to our hypotheses, patients with recurrent depressive episodes did not show significant regional gray-matter reductions compared to patients with first depressive episode.

According to our hypotheses, there were no gray-matter volume increases in patients with MDD detectable.

#### Region of interest analyses of the limbic system

**Figure 2:**
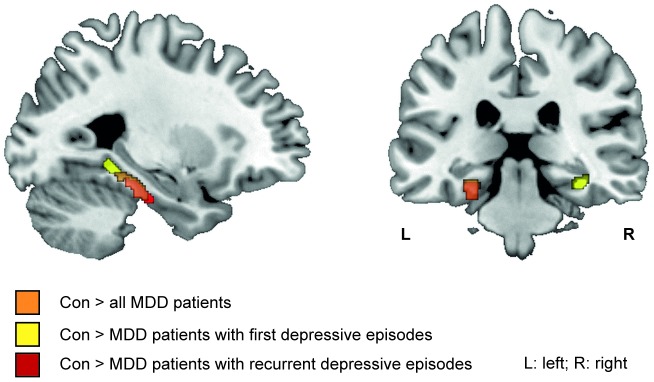
Gray matter volume reductions in the region-of interest (ROI) parahippocampal gyrus+hippocampus bilaterally. Gray matter volume reduction in ROI gyrus+hippocampus bilaterally in all MDD patients versus healthy controls (orange), patients with first depressive episode versus healthy controls (yellow) and patients with recurrent depressive episodes versus healthy controls (red) (Table 4). (Region-of-interest analyses, p<0.01, k = 109; view: MNI: −27 −29 −20).

According to our hypothesis, patients with first as well as with recurrent depressive episodes showed gray matter volume reductions in the hippocampal formation. Patients with recurrent episodes showed parahippocampal volume reductions in the left hemisphere, while in patients with first depressive episodes volume reductions were evident in both hemispheres (Table 4, Figure 2).

**Table 4:** Results of region-of-interest (ROI) analyses of the ROI hippocampus + parahippocampal gyrus bilaterally.

Contrast	Anatomical region	Side	Cluster size	MNI Coordinates (mm)			Z-Score	p-value (uncorr.)
				x	y	z		
**A) Con>Pat**	Parahippocampal gyrus	L	288	−27	−30	−20	3.71	<0.001
**B) Con>Pat with first depressive episode**	Parahippocampal gyrus	R	308	34	−36	−11	3.65	<0.001
	Parahippocampal gyrus	L	125	−26	−43	−6	3.02	<0.001
**C) Con>Pat with recurrent depressive episodes**	Parahippocampal gyrus	L	186	−27	−28	−23	3.26	0.001

Contrasts: A) MDD patients (n = 132) versus healthy controls (n = 132), B) patients with first depressive episode (n = 35) versus healthy controls (n = 35), C) patients with recurrent depressive episodes (n = 97) versus healthy controls (n = 97) and D) patients with first depressive episode (n = 35) versus patients with recurrent depressive episodes (n = 97). Analyses where conducted at p<0.01, uncorrected, k = 109 voxels.

ROI: region of interest.

MDD: major depressive disorder.

Con: controls; Pat: patients.

L: left; R: right.

In contrast to our hypothesis, ROI analyses of ACC and amygdala revealed no significant gray-matter volume reductions in patients with MDD.

### VBM-analyses: clinical correlations

**Figure 3:**
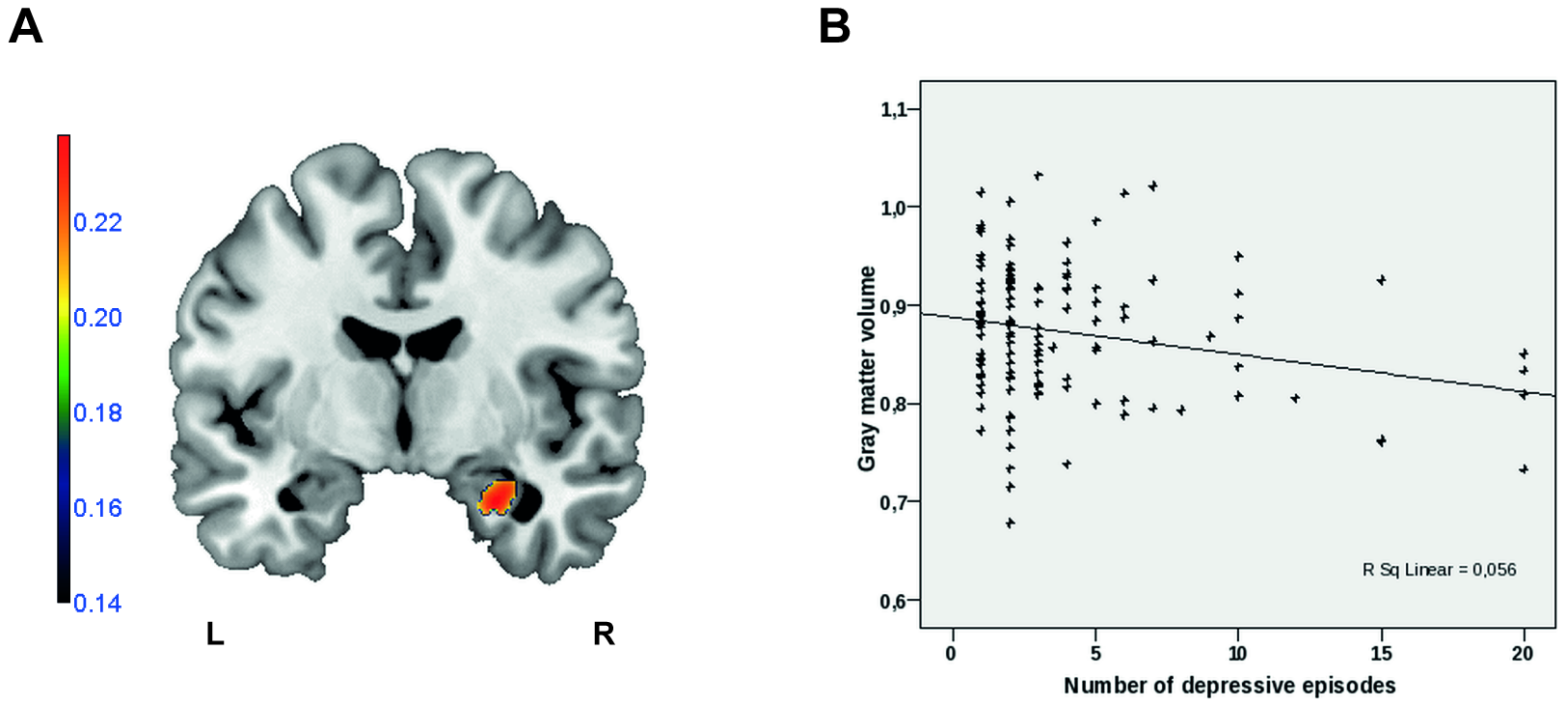
Number of depressive episodes is negatively correlated with the right hippocampal gray matter volume. **A**: Sagittal view (MNI x  = −6) depicting gray matter volumes correlating with number of depressive episodes. (Region-of-interest analyses, p<0.01, k = 109; Color bar represents negative correlation coefficient -r. (L: left; R: right)). **B**: Scatter plot depicting the negative correlation (r = −0.237; p = 0.006) of the right hippocampal cluster values (left panel) and the number of depressive episodes (SSPS Statistics 15.0 software package).

In the whole brain analysis, the number of depressive episodes was negatively correlated with the gray-matter volume in the right superior frontal gyrus (p<0.001, k = 139) (Table 5). According to our hypotheses, ROI analyses (Table 5) revealed significant negative correlations of the number of depressive episodes and gray-matter volume in the right hippocampus (p = 0.003, k = 109) (Figure 3) as well as in the right amygdala (p = 0.004, k = 10).

**Table 5:** Negative correlation of gray matter volume with increased number of depressed episodes in whole brain analysis and region-of-interest (ROI) analyses of the ROI hippocampus + parahippocampal gyrus bilaterally and of the ROI amygdala bilaterally.

	Anatomical region	Side	Cluster size	MNI Coordinates (mm)			Z-Score	p-value (uncorr.)
				x	y	z		
**Whole brain analysis**	SFG	R	180	24	12	63	3.36	<0.001
**ROI parahippocampal gyrus + hippocampus bilateral**	Hippocampus	R	153	26	−6	−24	2.73	0.003
**ROI amygdala bilateral**	Amygdala	R	29	30	−4	−20	2.67	0.004

Analyses where conducted at p<0.001, uncorrected, k = 139 voxels for whole brain analysis, k = 109 voxels for ROI hippocampus + parahippocampal gyrus bilaterally, k = 10 for ROI amygdala bilaterally.

ROI: region of interest.

SFG: superior frontal gyrus.

L: left; R: right.

ROI analyses of ACC revealed no significant negative correlations of the number of depressive episodes and gray-matter volume.

Exploring the influence of depression severity, there was a negative correlation between HDRS-score and gray-matter volume in the right postcentral gyrus (p<0.001; k = 291). In contrast, no significant correlation between BDI and gray-matter volume was observed.

In comparison to patients without anxiety comorbidities, patients with anxiety comorbidities showed gray-matter volume reductions in the right postcentral gyrus (p<0.001; k = 171).

Excluding patients with lithium medication from the group comparison between patients with MDD and healthy controls did not change the results. There were no significant differences in regional gray matter volume between patients with and without antipsychotic medication.

## Discussion

In this study we used VBM to investigate gray-matter volume differences between a large group of patients with unipolar major depression and a well-matched healthy control group and related our findings to clinical characteristics such as number of depressive episodes. In comparison to other studies with comparable samples sizes [33], [48], all patients were investigated in one institution with the same 3T-scanner and under identical measurement conditions. As one particularity of this study, analysis of patient sub-groups, e.g. patients with first depressive episodes and patients with recurrent episodes within the same sample, was performed. We could demonstrate gray matter volume reductions in insular as well as limbic regions.

In the present study, the most significant gray-matter reductions in patients with MDD in comparison to healthy controls were identified in the right anterior insula. The majority of the studies, investigating insula morphology [16], [18], [19], including our own, reported gray-matter reductions in the anterior parts of the insula. Importantly, the laterality of gray-matter reduction of the insula varies across these studies. A majority of studies describe volume reductions of the left insula [16]–[18], while others report bilateral volume reductions [19], [49] or volume reductions in the right hemisphere [50]. Possible explanations for these different findings could be differences in sample compositions. For example, insular gray-matter volume might be differently affected in first versus recurrent episode depression. In our study, we could demonstrate that the observed gray-matter reduction in the right anterior insula is based on volume reductions in patients with recurrent depressive episodes, but not in patients with first depressive episode. However, there does not seem to be a linear correlation between the number of depressive episodes and insular gray-matter volume, which goes in line with other studies [18]. In contrast, in a study of Lee and colleagues [49], illness duration, which was not assessed here, was negatively correlated with gray-matter volume in the left insula. Takahashi [18] reported moderate insular cortex volume reductions in patients with first depressive episode. Taking into account the small sample size of patients with first depressive episode in our study (n = 35), we cannot definitively exclude possible associations of the insular gray-matter volume with early stages of illness. Concerning the effect of current depression severity, Sprengelmeyer [16] reported a negative correlation between HDRS and BDI and bilateral insular volumes, while Takahashi [18] and ourselves could not show significant correlations between insular gray-matter volume and clinical measurements like HDRS and BDI. Our findings suggest that structural changes measured by VBM occur in the course of recurrent depressive episodes and do not reflect short-term clinical characteristics.

In conclusion there is accumulating evidence that insular gray matter might be altered in major depression. The insula cortex plays an important integrative role in processing of information from diverse functional systems [51]–[57]. Cytoarchitectonic studies as well as functional MRI and connectivity data indicate a set of anatomically and functional different regions within the insula [55], [57]–[63]. The anterior insula is involved in social-emotional and cognitive networks [55], connecting to the middle and inferior frontal cortex and to the anterior cingulate cortex (ACC) [61]. There is growing evidence that these connections might play an integrative role in cognitive, affective and behavioral contexts [52], thus all domains frequently affected in depressive disorders. Reduced insular and ACC-volumes as well functional changes in these regions are recurrent imaging findings in MDD [3], [64]. The insular plays an important role in emotion processing and has been assigned a pivotal role in Damasio's theory of emotions. Accordingly, emotions are cerebral representations of bodily states. There is a neural network within the insula mediating emotionally relevant information from spinal cord and vagus afferents via the posterior (VMpo) and basal part (VMb) of the ventromedial nucleus of the thalamus to the ventromedial and frontal cortex as well as to anterior cingulate [65]. Insula volume loss may therefore reflect impaired insular function and connectivity, related to depressive symptomatology. Considering functional imaging studies, there is accumulating evidence that the insular cortex, especially the right anterior insula, might be an important structure involved in MDD [15]. Our findings suggest that gray-matter reduction of the right anterior insula in patients with MDD is associated with a recurrent course of illness. The question, if volumetric changes of the insular lobe are trait- or state markers, needs to be addressed in further studies.

Another key finding in our study was a gray-matter reduction in the hippocampus formation in patients with MDD in comparison to healthy control subjects. These results are consistent with other studies reporting parahippocampal gray-matter volume reductions [11], [16], [30], [31]. Reduced hippocampal volume is a frequently reported structural abnormality in major depressive disorder [66], [67] while only a minority of studies does not share this observation [68]–[71]. Meta-analyses confirmed that in the aggregate the hippocampus is reduced in patients with major depression [66], [67], [72], [73]. Study results differ in localization of hippocampal volume loss with studies showing bilateral [11], [28], [49], [74]–[77], left unilateral [30], [78]–[80] and right unilateral [31], [81], [82] atrophy, which might be due to potential influencing factors like age, illness duration, recurrences, illness severity, comorbidity, medication or definition of hippocampal borders in MR images [83]. With respect to clinical variables influencing hippocampal volume, findings are inconsistent [67], [84]. In a meta-analysis, McKinnon et al [67] could demonstrate that decreased hippocampal volumes were apparent only in patients with more than one depressive episode or with an illness duration of more than 2.5 years. In contrast, there are also studies reporting hippocampal atrophy in first episode depression [66], [80], [85] and even in healthy subjects at high risk for depression [86]–[88] and with early life stress [89], [90]. In the present study we could demonstrate that decreased gray-matter volume in the hippocampal formation was evident already in patients with a first depressive episode as well as in patients with recurrent depressive episodes in comparison to healthy controls. Furthermore we could demonstrate a negative correlation between the gray-matter volume of the right hippocampus and the number of depressive episodes. This finding is consistent with other studies describing inverse correlations between hippocampal gray-matter volume and illness duration/number of depressive episodes [91]–[94]. Our study results support the idea that hippocampal volume reductions and long illness duration [91], [95] respectively duration of untreated depressive symptoms [96] may be related. This is in accordance with the neurotoxicity hypothesis of hippocampal atrophy in MDD, explaining hippocampal volume reduction by prolonged exposure to stress-induced biochemical abnormalities, mediated e.g. via HPA-axis, BDNF, or inflammatory processes [46], [97]. Arnone et al [96] reported gray matter increase in the hippocampus following acute treatment and remission. The study results are consistent with preclinical studies showing that neuroplastic changes in the hippocampus follow antidepressant treatment [98], [99]. Comparable results have been reported after successful treatment with electroconvulsive therapy [100]. Taken together, there is growing evidence, that hippocampal atrophy is state dependent.

An alternative explanation for hippocampal atrophy in MDD is provided by the vulnerability hypothesis, suggesting that hippocampal atrophy is a pre-existing risk factor for MDD [97] and is therefore already evident in first depressive patients. A limitation of our study is the lack of information about early life stress and genetic information. Therefore we could not test the influence of these factors on hippocampal volume in patients and healthy controls.

Hippocampal volume reductions seem to be clinically relevant. In several studies [30], [68], [80], [91], [101]–[103], larger hippocampal volumes were associated with better clinical responses/remission and with lower relapse rates in comparison to patients with small hippocampal volumes. Therefore small hippocampal volumes may predict poor treatment outcome and increased risk of relapse of depression [84], which is extremely relevant in assessing the further prognosis. As part of the limbic system and limbic-cortical-striatal-pallidal-thalamic networks [84], the hippocampus plays an important role in memory-related cognitive processes as well as in motivation and emotion [75], [76]. Hippocampal atrophy may therefore reflect impaired hippocampal function and connectivity, related to depressive symptomatology [75], [76], [84]. In conclusion, our results of hippocampal volume loss in patients with first as well as in patients with recurrent depressive episodes and the finding of an inverse correlation of number of depressive episodes and hippocampal volume reduction support the idea of a combination of the neurotoxicity and vulnerability hypothesis as an explanatory model for hippocampal volume loss in patients with MDD.

The amygdala is another important structure of the limbic system, closely related to the hippocampus. In the present study we found a negative correlation between gray-matter volume of the right amygdala and number of depressive episodes. On the other hand, we could not find reduced gray-matter volume of the amygdala in patients with MDD at group comparison level. Previous volumetric studies of the amygdala showed inconsistent results with studies reporting unchanged [104]–[106], increased [105], [107]–[110] as well as reduced volumes [69], [94], [111]–[114] in patients with MDD. Also meta-analyses showed contradictory findings [3]–[5], [115]. These discrepancies may be due to different clinical characteristics of the samples, such as medication [116], depression severity [94], [109], or illness duration/number of depressive episodes [3], [105], [107], [108], [114]. Our finding of an inverse correlation of number of depressive episodes and amygdala volume goes in line with study results of Kronenberg et al. [114] and may be an indicator that depression decreases amygdala volume [114]. In contrast, other authors did not report this association [69], [105], [117]. In the absence of significant differences at group comparison level, interpretation of our data should be taken very carefully and remain to some degree speculative. Therefore, it is still unclear, if volumetric changes in the amygdala are trait or state-markers. Longitudinal studies are needed to answer this question.

### Methodological limitations

Brain-imaging analyses usually suffer from limitations in patients comparabilities. Heterogeneity of and even in the studied samples is one of the main problems in the inconsistency of MRI findings in depression. As mentioned in the introduction, there are diverse confounding factors. Especially the influences of co-morbidity and of the mostly unique medication history are hard to control and are likely to confound results. This has to be kept in mind, when interpreting study results. On the other hand, recruiting a sample of drug-naïve patients without any co-morbidity leads generally to small sample sizes.

A good approach for controlling possible pharmacological effects is to consider the cumulative drug dose that patients were exposed to prior to the MRI scan. To improve comparability of medication, equivalent dosages can be calculated. In terms of antipsychotic medication, chlorpromazine equivalence is an often used approach [118], but also other calculated (e.g. maximum dose and daily-defined dose,) and consensus approaches exist [118]. In terms of antidepressant medication, a classification of Sackeim et al. [119] is frequently used. As mentioned above, in the study presented, we included a large sample of patients with a great heterogeneity in medication. Stratification according to single antidepressants would have resulted in small subgroups. Unfortunately, medication history in many patients remained vague, thus calculation of cumulative drug dose would have been too speculative. Therefore we cannot comment on pharmacological effects and specific studies on medication effects on brain volume are needed. It is known that antidepressants may exert neurotrophic or neuroprotective effects [120]–[124]. However, this effect would lead to volume increases in patients and might have attenuated, but not caused the volume loss observed here. Nevertheless, excluding patients with lithium medication did not change our results. In addition, there were no significant differences in regional gray matter volume between patients with and without antipsychotic medication.

Comorbid disorders were carefully considered here. Comorbidity of depression and anxiety disorder is frequent in MDD [125]–[128]. In a VBM-study, van Tol [33] could demonstrate similar as well as different gray-matter volume differences in patients with and without anxiety disorder in comparison to healthy control subjects. In our study, the only structure differing between patients with and without anxiety disorder comorbidity was a gray-matter reduction in the right postcentral gyrus in patients with anxiety disorders.

A limitation of our study is the lack of information about early life stress and genetic information as well as psychiatric family history. These are important information to differ between state- and trait-dependent changes. We cannot exclude possible influences of these lacking information on brain volumes of healthy controls included in this study. Further studies are needed, including subjects who are at risk for MDD, for example relatives of MDD patients and persons with a history of abuse and stressful early life events.

Generally, the interpretation of results of this study and any VBM-based analysis should be taken with care. VBM cannot offer direct information about the underlying cellular mechanism mediating the observed effects. It is nonspecific with respect to the underlying tissue and does not provide information on the molecular and cellular mechanisms of the analyzed brain regions. Phenomena visible with VBM are never the result of a single process; moreover there are the result of a multifactorial process including multiple cellular modifications, for example cell density, cell size, myelination and vascularization. All these factors affect relaxation times and therefore influence voxel intensities on a T1-weighted image [129]. The exact molecular and cellular mechanisms of volumetric changes during the course of depressive disorder and their influences on brain function still remain unclear. Therefore deductive reasoning from volumetric changes to functional changes remains speculative to some degree, including the distinction of state versus trait markers [96].

Taking into account the mentioned limitations, studies combining the use of different neuroimaging techniques (VBM, DTI, fMRI) in combination with more precise anatomical techniques (e.g. manual or automated segmentation [83], measurement of cortical thickness, fiber tracking) and in combination with molecular techniques like MR-spectroscopy are needed. Taking into account possible influences and structural and functional brain changes over the course of depressive disorder, longitudinal studies are needed. In this connection, neuroimaging techniques offer the advantage, that they can be repeatedly performed in the same individual.

The strength of the present study is its large sample size and subgroup analyses of patients with first depressive episode as well as with recurrent episodes. We could enforce results from previous studies reporting insular and hippocampal gray matter volume reductions in patients with major depressive disorder.

## Conclusions

Our findings indicate that the right anterior insular anatomical structure is strongly affected in MDD and is associated with a recurrent course of illness. This strong insular volume loss may reflect impaired insular function and might therefore play an important role in the neurobiology of depression. The hippocampal and amygdala volume loss cumulating with the number of episodes might be explained either by repeated neurotoxic stress or alternatively by higher relapse rates in patients showing hippocampal atrophy.

## Supporting Information

Table S1 List of pharmacological treatment including antidepressants, antipsychotics, lithium and mood stabilizers.
(DOCX)
