# Supplementary material for: Insular and Hippocampal Gray Matter Volume Reductions in Patients with Major Depressive Disorder
Source: PLoS One. 2014 Jul 22;9(7):e102692. doi: 10.1371/journal.pone.0102692 (PMC4106847; doi:10.1371/journal.pone.0102692)
Supplement: Table S1 — List of pharmacological treatment including antidepressants, antipsychotics, lithium and mood stabilizers. (DOCX) [file pone.0102692.s001.docx]

**Table S1** List of pharmacological treatment

|  | **MDD first depressive episode**  **n=35** | **MDD recurrent depressive episodes**  **n=97** |
| --- | --- | --- |
| **Antidepressive monotherapy** |  |  |
| Agomelatine | 2 | 0 |
| Mirtazapine | 4 | 12 |
| NDRI | 0 | 1 |
| SNRI | 1 | 0 |
| SSNRI | 7 | 23 |
| SSRI | 7 | 16 |
| TZA | 0 | 1 |
| **Antipsychotic monotherapy** |  |  |
| Quetiapine | 0 | 1 |
| **Combined antidepressive therapy** |  |  |
| Mirtazapine+SSRI | 2 | 3 |
| SNRI+SSRI | 0 | 2 |
| SSNRI+Mirtazapine | 2 | 10 |
| SSNRI+Mirtazapine+Agomelatine | 1 | 0 |
| SSNRI+Mirtazapine+SSRI | 0 | 1 |
| **Combined antidepressive/antipsychotic therapy** |  |  |
| Mirtazapine+Quetiapine | 0 | 1 |
| Mirtazapine+SSRI+Quetiapine | 1 | 1 |
| Mirtazapine+SSRI+Quetiapine+Pipamperone | 1 | 0 |
| SNRI+Olanzapine | 0 | 1 |
| SSNRI+Mirtazapine+Quetiapine | 0 | 2 |
| SSNRI+Quetiapine | 0 | 4 |
| SSNRI+Risperidone | 0 | 1 |
| SSNRI+SNRI+Quetiapine | 1 | 0 |
| SSNRI+SSRI+Olanzapine | 0 | 1 |
| SSNRI+SSRI+Pipamperone | 1 | 0 |
| SSNRI+Ziprasidone+Pipamerone | 0 | 1 |
| SSRI+Quetiapine | 0 | 1 |
| TZA+Amisulpride | 1 | 0 |
| TZA+Olanzapine | 0 | 1 |
| **Lithium** |  |  |
| Mirtazapine+SNRI+Olanzapine+Risperidone+Lithium | 0 | 1 |
| Quetiapine+Lithium | 0 | 1 |
| SSNRI+Lithium | 1 | 0 |
| SSNRI+Olanzapine+Lithium | 1 | 0 |
| SSNRI+Quetiapine+Lithium | 0 | 1 |
| SSNRI+Risperidone+Lithium | 0 | 1 |
| **Lithium+Mood Stabilizers** |  |  |
| NDRI+Lithium+Lamotrigin | 0 | 1 |
| TZA+Promethazin+Lithium+Topiramat | 0 | 1 |
| **Mood Stabilizers** |  |  |
| Mirtazapine+SSNRI+Quetiapine+Lamotrigin | 0 | 1 |
| MAOH+Quetiapine+Lamotrigin | 0 | 1 |
| SSNRI+Mirtazapine+Lamotrigin | 0 | 1 |

*NDRI = Selective noradrenaline-dopamine-reuptake inhibitors*

*SNRI = Selective noradrenaline-reuptake-inhibitors*

*SSNRI=Selective serotonin-noradrenaline-reuptake-inhibitors*

*SSRI = Selective serotonin-reuptake inhibitors*

*TZA = Tricyclic antidepressants*

*MAOH = Monoamine oxidase inhibitors*
